# Interactions between *Nosema* microspores and a neonicotinoid weaken honeybees (*Apis mellifera*)

**DOI:** 10.1111/j.1462-2920.2009.02123.x

**Published:** 2010-03

**Authors:** Cédric Alaux, Jean-Luc Brunet, Claudia Dussaubat, Fanny Mondet, Sylvie Tchamitchan, Marianne Cousin, Julien Brillard, Aurelie Baldy, Luc P Belzunces, Yves Le Conte

**Affiliations:** 1 INRA, UMR 406 Abeilles et Environnement, Laboratoire Biologie et Protection de l'abeille, Site Agroparc84914 Avignon, France; 2 INRA, UMR 406 Abeilles et Environnement, Laboratoire de Toxicologie Environnementale, Site Agroparc84914 Avignon, France; 3 INRA, UMR 408 Sécurité et Qualité des Produits d'Origine Végétale, Site Agroparc84914 Avignon, France

## Abstract

Global pollinators, like honeybees, are declining in abundance and diversity, which can adversely affect natural ecosystems and agriculture. Therefore, we tested the current hypotheses describing honeybee losses as a multifactorial syndrome, by investigating integrative effects of an infectious organism and an insecticide on honeybee health. We demonstrated that the interaction between the microsporidia *Nosema* and a neonicotinoid (imidacloprid) significantly weakened honeybees. In the short term, the combination of both agents caused the highest individual mortality rates and energetic stress. By quantifying the strength of immunity at both the individual and social levels, we showed that neither the haemocyte number nor the phenoloxidase activity of individuals was affected by the different treatments. However, the activity of glucose oxidase, enabling bees to sterilize colony and brood food, was significantly decreased only by the combination of both factors compared with control, *Nosema* or imidacloprid groups, suggesting a synergistic interaction and in the long term a higher susceptibility of the colony to pathogens. This provides the first evidences that interaction between an infectious organism and a chemical can also threaten pollinators, interactions that are widely used to eliminate insect pests in integrative pest management.

## Introduction

The current decline in abundance and diversity of wild bees as well as honeybees has been reported in several regions of the world (Biesmeijer *et al.*, 2006; National Research Council of the National Academies, 2007). The magnitude of this pollinator crisis is believed to not only have a deep impact on agriculture and its related economy (Gallai *et al.*, 2009) but also on plant diversity (Biesmeijer *et al.*, 2006) and landscapes (Ricketts *et al.*, 2008). The most spectacular pollinator decline concerns honeybee colonies, which are disappearing en masse in USA and Europe (Faucon *et al.*, 2002; Higes *et al.*, 2005; Oldroyd, 2007; Stokstad, 2007). Although many stressors have been identified as a potential cause or indicator of colonies losses, including viruses (Cox-Foster *et al.*, 2007), microsporidia pathogens (Higes *et al.*, 2008; 2009;) and pesticides (Frazier *et al.*, 2008), a combination of multiple agents is more likely to contribute to honeybee losses. Therefore, investigations have to be carried out on integrative effects of different agents.

A large spectrum of pesticides is used to manage crop pests. But as an alternative, and to reduce the harmful effects of chemicals on non-pest organisms and human, new eco-friendly strategies for controlling crop pests have been developed. These biological controls include the use of microbial pathogens like viruses, bacteria and fungi. Modern crop management integrates these different techniques in a compatible manner leading to an integrated pest management (IPM) (Maredia *et al.*, 2003). The most extensively used biological agents are fungi, which are often associated with insects [around 750 species are pathogens of insects (Carruthers and Soper, 1987)]. Entomopathogenic fungi and chemical insecticides used together significantly improve the lethality of control agents. Indeed, when fungi are delivered with sub-lethal doses of pesticides, they interact synergistically in killing insects (Purwar and Sachan, 2006). Among the insecticides, the neonicotinoid imidacloprid is one of the most effective in interacting synergistically with fungi. And IPM using the synergy between imidacloprid and fungal spores is commonly used for killing a variety of insect pests, like termites, thrips and leaf-cutter ants (Ramakrishnan *et al.*, 1999; Al Mazraáwi, 2007; Valmir Santos *et al.*, 2007).

Interestingly, imidacaloprid is a systemic insecticide widely used worldwide on food crops and has been believed to cause honeybee losses in France (Doucet-Personeni *et al.*, 2003). Despite a high percentage of hives containing residues of imidacloprid [e.g. in France, more than one hive in two has residues of imidacloprid and its metabolite 6-chloronicotinic acid in the pollen, 30% in honey and 26% in bees (Chauzat *et al.*, 2009)], the level of exposure is sub-lethal with no obvious effect on mortality (Schmuck *et al.*, 2001; Nguyen *et al.*, 2009). On the other side, a parasitic microsporidia, *Nosema ceranae*, has been associated to bee losses in USA without contributing significantly to it (Cox-Foster *et al.*, 2007), but it is reported to be a cause of bee losses in Spain (Higes *et al.*, 2008; 2009;).

Ironically, the combination of pathogens and pesticides that may be effective for insect pest control may result specifically in imidacloprid and *Nosema* acting together to kill bees. Because a single factor would not explain honeybee or more generally pollinator decline, it is highly possible that stressors act in concert. So, we ask the question of whether honeybees are victim of an interaction between infectious organism and a chemical like in IPM.

We looked at interactive effects between biological and chemical stressors on pollinators by analysing the interaction between imidacloprid and *Nosema* in honeybees. As social organisms, honey bees depend not only on the health of individuals, but also on the overall functioning of the hive. Consequently, we tested those integrative effects on honeybee health, at two levels, the individual and colony level. This study was designed to look at a possible effect on: (i) individual mortality and energetic demands; (ii) individual immunity; and (iii) social immunity. Sucrose consumption was calculated to estimate the energetic stress as *Nosema* alters host nutrient store and feeding behaviour (Mayack and Naug, 2009; Naug and Gibbs, 2009). Total haemocyte count (THC) and phenoloxidase (PO) enzymatic activity were analysed as parameters of individual immunity. Phenoloxidase plays a central role in invertebrates' immune reaction, being implicated in the encapsulation of foreign object through melanization (Decker and Jaenicke, 2004). Total haemocyte count gives an indirect measurement of basal cellular immunocompetence and is involved in the processes such as the phagocytosis and the encapsulation of a parasite (Tanada and Kaya, 1993). Those two defence reactions have been observed against fungal pathogens in insects (Charnley, 1984). Finally, glucose oxidase (GOX) enzymatic activity was analysed as a parameter of social immunity. Mainly expressed in the hypopharyngeal glands (HPGs) (Ohashi *et al.*, 1999), GOX catalyses the oxidation of β-d-glucose to d-gluconic acid and hydrogen peroxide, the latter having antiseptic properties (White *et al.*, 1963). The antiseptic products are secreted into larval food (Sano *et al.*, 2004) and into honey (White *et al.*, 1963; Ohashi *et al.*, 1999) which contributes to colony-food sterilization and therefore to diseases prevention. Indeed, the level of hydrogen peroxide in honey is positively correlated with the inhibition of pathogens development (Taormina *et al.*, 2001; Brudzynski, 2006).

## Results

### Effect of *Nosema* infection and/or exposure to imidacloprid on bee mortality and energetic demand

**Fig. 1:**
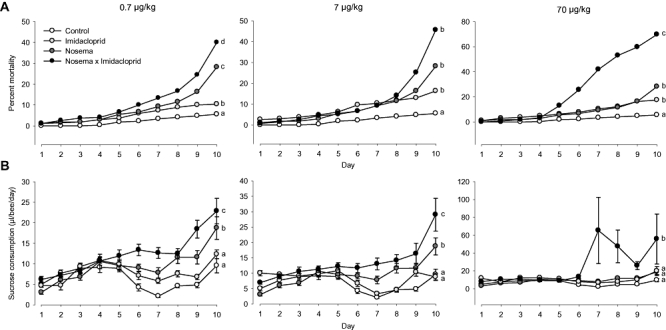
Effect of *Nosema* infection and/or exposure to imidacloprid on bee mortality and energetic demands. A. Effect on mortality. Mortality is expressed as the percentage of cumulated number of dead bees per cage and per day (*n* = 270 bees). Three colonies were analysed, with three cage replicates for each colony (*n* = 30 bees per cage). Each letter indicates significant differences between treatments (*P* < 0.05). B. Effect on energetic demand. Sucrose consumption is expressed as the amount of sucrose solution (50% w/v, *ad libitum* delivery) consumed per day and per bee (*n* = 30 bees per cage) during the 10 h of treatment. The same cages as in A were analysed. Each letter indicates significant differences between treatments (*P* < 0.05).

The cumulative mortality rate increased with time in all experimental groups, but remained lower in control groups (∼5%) (*P* < 0.001 for each imidacloprid concentration, Fig. 1A). In addition, an important treatment effect was detected (*P* < 0.001 for each imidacloprid concentration). Indeed, all three treatment groups exhibited significantly higher mortality rates than the control group (Fig. 1A). The effect of *Nosema* infection and imidacloprid exposure did not differ significantly except for the low concentration of imidacloprid (Fig. 1A). For each imidacloprid concentration, the mortality was the highest in bees when also challenged with *Nosema*. Interestingly, on the last 2 days of rearing, mortality rates of the *Nosema* × imidacloprid group equalled the sum of the mortality rates of the *Nosema* and imidacloprid groups, showing an additive effect, which was significant for the low imidacloprid concentration. The interactive effect was even stronger with the high concentration of imidacloprid showing, in that case, a potentiating effect.

The sucrose consumption measurements, which were performed on the same cages as those used for the mortality assay, showed a similar pattern to the mortality rate. The amount of sucrose solution consumed significantly increased with time (*P* < 0.001 for each imidacloprid concentration, Fig. 1B) and was affected by the treatments (*P* < 0.001 for each imidacloprid concentration, Fig. 1B). Bees infected with *Nosema* consumed significantly more sucrose than control and imidacloprid-exposed bees. This amount was the highest in bees both infected with *Nosema* and exposed to imidacloprid (Fig. 1B).

**Fig. 2:**
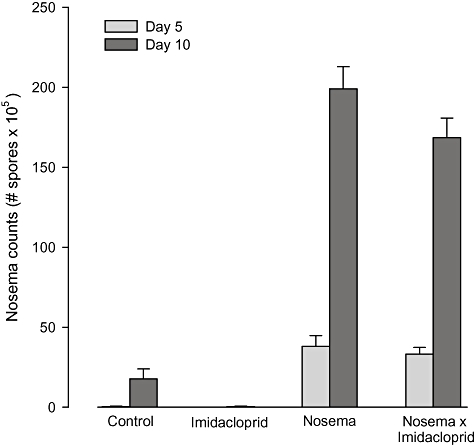
Level of *Nosema* infection in bees fed with *Nosema* and/or exposed to imidacloprid. Level of infection was determined at days 5 and 10 on seven to eight bees per cage for each experimental group (*n* = 382 bees). Three colonies were analysed, with two cage replicates for each colony. Data show mean ± SE.

The number of *Nosema* spores also increased with time even in the control groups, meaning that some control bees were likely infected at the beginning of the experiment (Fig. 2). However, the level of *Nosema* infection was significantly different between bees fed with *Nosema* (*Nosema* groups and *Nosema* × imidacloprid groups) and control bees or bees only exposed to imidacloprid (*P* < 0.001 for each comparison). Interestingly, at day 10, bees exposed to imidacloprid had a slightly lower number of spores than bees non-exposed to imidacloprid suggesting a slight inhibiting effect of imidacloprid on spore germination; a difference that was marginally significant between groups of bees individually infected with *Nosema* (control versus imidacloprid: *P* = 0.11, *Nosema* versus *Nosema* × imidacloprid: *P* = 0.051).

### Effect of *Nosema* infection and/or exposure to imidacloprid on individual immunity

**Fig. 3:**
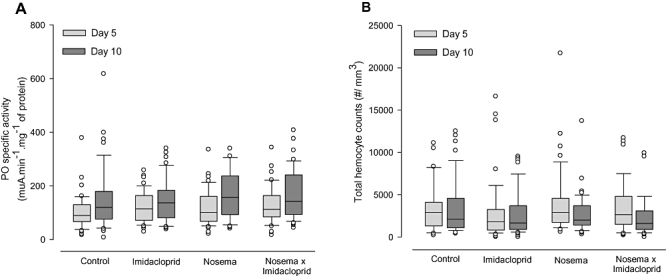
Effect of *Nosema* infection and/or exposure to imidacloprid on individual immunity. A. Total haemocyte counts at days 5 and 10 on seven to eight bees per cage for each experimental group (*n* = 373 bees). B. Phenoloxidase activity at days 5 and 10 in eight bees per cage for each experimental group (*n* = 384 bees). For each parameter, three colonies were analysed, with two cage replicates for each colony. Boxes show 1st and 3rd interquartile range with line denoting median. Whiskers encompass 90% of the individuals, beyond which each outliers are represented by circles.

Phenoloxidase enzymatic activity was normalized to the protein concentration, which did not differ between experimental groups and age but changed between colonies (*F*_1,388_ = 1.06, *P* = 0.31; *F*_3,388_ = 1.88, *P* = 0.13; *F*_2,388_ = 8.75, *P* < 0.001 respectively). Phenoloxidase specific activity was not affected by *Nosema* infection and/or exposures to imidacloprid (Fig. 3A). Similarly, THC did not change between the different groups (Table 1, Fig. 3B). However, PO-specific activity and THC were found to, respectively, increase and decrease with age as found by Schmid and colleagues (2008) and Wilson-Rich and colleagues (2008) (Table 1, Fig. 3A and B). There was also a significant variation between colony replicates (Table 1).

**Table 1:** Analysis of *Nosema* infection, individual (THC, PO) and social immunity (GOX, HPG) as a function of experimental treatment (control, *Nosema*, imidacloprid and *Nosema* × imidacloprid), age and colony origin.

Parameter	Source of variation	d.f.	*F*	*P*
*Nosema*	Treatment	3, 358	161.3	< 0.001
	Age	1, 358	265.5	< 0.001
	Colony	2, 358	10.9	< 0.001
THC	Treatment	3, 349	1.3	0.274
	Age	1, 349	5.4	0.021
	Colony	2, 349	13.9	< 0.001
PO	Treatment	3, 352	1.57	0.197
	Age	1, 352	10.9	< 0.001
	Colony	2, 352	17	< 0.001
GOX	Treatment	3, 182	4.6	0.004
	Colony	1, 182	1.9	0.168
HPG	Treatment	3, 180	7.3	< 0.001
	Colony	2, 180	1.2	0.288

### Effect of *Nosema* infection and/or exposure to imidacloprid on social immunity

**Fig. 4:**
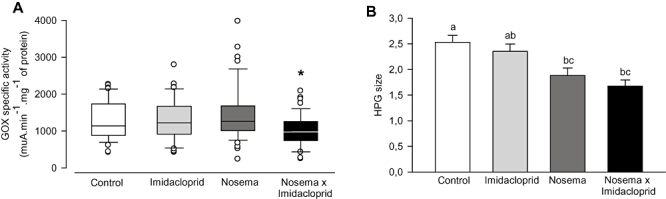
Effect of *Nosema* infection and/or exposure to imidacloprid on social immunity. A. Glucose oxidase activity at day 10 on eight bees per cage for each experimental group (*n* = 192 bees). Boxes show 1st and 3rd interquartile range with line denoting median. Whiskers encompass 90% of the individuals, beyond which each outliers are represented by circles. *denotes significant difference between *Nosema* × imidacloprid groups and the three others groups (*P* < 0.05). B. HPG size at day 10 in seven to eight bees per cage for each experimental group (*n* = 191 bees). For each parameter, three colonies were analysed, with two cage replicates for each colony. The size was indexed from 1 to 5 (see *Experimental procedures*). Each letter indicates significant differences between treatments (*P* < 0.05). Data show mean ± SE.

The protein concentration in the head changed significantly according to the treatments and colony origin (*F*_3,175_ = 5.78, *P* < 0.001; *F*_2,175_ = 36.9, *P* < 0.001 respectively). Bees from *Nosema* × imidacloprid groups had a lower protein concentration (4.4 ± 1.3 × 10^−3^ mg ml^−1^) than bees from the control (4.9 ± 1.2 × 10^−3^), *Nosema* (4.8 ± 0.9 × 10^−3^) and imidacloprid groups (4.8 ± 1.2 × 10^−3^) (*P* < 0.01, *P* < 0.01, *P* < 0.05 respectively). A significant effect of treatments on the specific activity of GOX was detected (Table 1, Fig. 4A). The combined effects of *Nosema* infection and exposure to imidacloprid significantly decreased the GOX-specific activity compared with control, *Nosema* and imidacloprid groups (*P* = 0.013, *P* < 0.001 and *P* < 0.01 respectively; Fig. 4A), demonstrating a synergistic effect between the two stressors. This response of GOX activity was highly consistent because there was no significant difference between colony replicates (Table 1).

The HPG size was also affected by the treatments (Fig. 4B). Bees from the *Nosema* × imidacloprid group possessed smaller HPG than control (*P* < 0.001) and imidacloprid-exposed bees (*P* = 0.004), but were not different from bees infected with *Nosema* (*P* = 0.27). Contrary to the GOX activity results, bees infected with *Nosema* had smaller HPG than control bees (*P* < 0.01) but they were not different from bees exposed to imidacloprid (*P* = 0.09). As for GOX activity, those differences were steady between colony replicates (Table 1).

## Discussion

Because current hypotheses about honeybee colony losses strongly suggest multifactorial causes, we addressed for the first time the effect of an interaction between a parasite and a pesticide on honeybee health. Our results demonstrated interactive effects between microsporidia and pesticides that weaken honeybee health.

Malone and Gatehouse (1998) observed that bees could ingest some spores by chewing the wax capping at emergence, which could explain the detection of some spores in control bees. This observation suggests that we compared lightly to heavily (experimentally) infected bees; however, the mortality rate in the first group was insignificant. Bees that were both infected with *Nosema* and exposed to imidacloprid at concentrations encountered in the environment showed the highest mortality rate. Interestingly, the sucrose feeding followed a similar pattern both regarding the treatment and time effect. This correlation gives some clues about the mechanisms of the interaction between *Nosema* and imidacloprid. *Nosema ceranae* can affect nutrient needs in hosts by using host nutrients and inducing an energetic stress (Mayack and Naug, 2009; Naug and Gibbs, 2009). Microsporidia are usually amitochondriate and unable to perform oxidative phosphorylation, meaning that they have a high dependency on host ATP (Keeling and Fast, 2002; Cornman *et al.*, 2009), especially for germination which requires high level of energy. However, microsporidian spores have retained the glycolytic pathway suggesting that they are able to use glycolysis to produce ATP (Keeling and Fast, 2002). This idea is supported by a significant drop in trehalose levels (glucose–glucose disaccharide) in hosts during the germination of *Nosema algerea* (Undeen and Vander Meer, 1994). In our study, this dependence on host energy triggered also an increase in sucrose needs in bees that are challenged by *Nosema* parasitism. Imidacloprid alone did not increase food intake, meaning that it is not particularly attractive to the bees. However, when the food was treated with imidacloprid, the boost in food intake caused by parasitism was associated with an increase in imidacloprid exposure. Although imidacloprid contamination in the hive is usually found at sub-lethal doses, microsporidia infection could have the capacity to expose bees to lethal doses by increasing the intake of contaminated food. This is particularly striking with the high concentration of imidacloprid used here, where *Nosema* and imidacloprid irremediably potentiates their effects.

Besides their direct impacts on host survival, pathogens can also impose significant costs on immunity. For example, one strategy of pathogens to promote their survival and replication in hosts is to suppress the activity of the immune system, which can involve the depression of PO activity (Yang and Cox-Foster, 2005) and haemocyte population (Ibrahim and Kim, 2006). However, our results showed that PO activity was neither up- nor down-regulated by *Nosema* challenge alone or in combination with imidacloprid. Similarly, THC was not affected by the different treatments. Antunez and colleagues (2009) showed that *Nosema apis* induced a higher expression of the gene coding for PO, but at the enzymatic level, we did not observe higher activity. The lack of immune response might be explained by deficient immunoregulatory activation, a lack of stimulation by microsporidia, or both. However, we cannot exclude that other parameters of individual immunity were activated or immunosuppressed, like antibacterial peptides and other immunity-related enzymes (e.g. glucose dehydrogenase, lyzozyme) (Antunez *et al.*, 2009).

Another type of immunity that can be found in social insects and particularly in honeybees is a social immunity, which consists in a cooperation between the individual group members to prevent disease contamination (Cremer *et al.*, 2007; Wilson-Rich *et al.*, 2009). The analysis of the honeybee genome showed that honeybees possess only one-third the number of immune response genes known for solitary insects (Evans *et al.*, 2006). This apparent lack of immune genes could be explained by a highly effective and maybe less costly social immunity compared with individual immunity (Cremer *et al.*, 2007). In honeybees, collective immune defence is well developed and includes hygienic behaviour, which is an antiseptic behaviour consisting of the ability to detect and remove diseased brood from the hive (Wilson-Rich *et al.*, 2009). The secretion of antiseptics in brood food and honey constitute another type of social immunity. Interestingly, the interaction between parasitism and exposure to pesticides induced an immunosuppression at the social level by causing a significant decline of GOX activity. This enzyme is essential in producing the antiseptic and thus sterilizing larval food (Sano *et al.*, 2004) and honey (White *et al.*, 1963; Ohashi *et al.*, 1999). As a result, if the colony is not able to maintain levels of GOX activity by recruiting more workers for this task, a reduction of antiseptics in the colony would not only affect adult nestmates but also the brood survival, i.e. would weaken the colony in the long term. And even if the colony responds accurately to the need for antiseptic production by a massive worker recruitment, this would reduce worker allocation in others tasks (like food collecting) and thus induce also a cost for the colony.

The mechanisms by which the combination of both stressors causes a reduction in GOX activity are not known. Glucose oxidase is mainly expressed in the HPG (Ohashi *et al.*, 1999), but the size reduction of HPG observed in bees infected with *Nosema*, as also found by Wang and Moeller (1969), is not associated with a decline in GOX activity, suggesting no link between HPG size and GOX activity. One possible explanation is that microsporidia use glucose to generate energy for their development (see above). As a result, the lack of glucose available to the bee could be followed by a decrease in the expression of GOX. However, the similar spore number in *Nosema* groups and *Nosema* × imidacloprid groups does not explain the depression in GOX activity in the last group. So it is reasonable to suppose that the interaction of both stressors might accentuate the energetic stress and induce a cost for GOX production that cannot be overcome.

In order to determine the consistency of our results, we conducted the experiments on three different colonies and observed that colony origin had a significant effect on PO activity and THC. The different responses between colonies could be explained by different colony environment history (pathogens, food sources), genetic background or both. However, the colonies that were used in the experiments came from the same location and were exposed to the same environment, suggesting that genetic variation might influence those individual immunity parameters. Indeed, Evans and Pettis (2005) found considerable genetic variation between colonies regarding the immune responsiveness of colony members. On the contrary, GOX activity was consistent between colonies, which would suggest a lower genetic variation across colonies regarding antiseptic production. A current hypothesis suggests that if social immunity is less costly and more effective than individual immunity, then selective pressure would favour collective defence against disease at the expend of individual defence (Cremer *et al.*, 2007). Consequently, higher selective pressure on social immunity would reduce genetic variation of this trait; however, this needs to be tested.

In summary, the interaction between microspore parasites and pesticide not only caused a higher rate of mortality but also demonstrated the potential to weaken colonies. By focusing either on the effects of pesticides or on parasites alone, their well-established interaction has been completely ignored despite clear evidences in IPM that entomopathogenic fungi act synergistically with sub-lethal doses of pesticides to kill insect pests. Thus, our study paves the way for future studies that will begin to tease apart the multiple factors that strain pollinator health. Therefore, multifactorial analysis should be performed in other pollinator' species such as bumblebees, which show similar sensitivity to pesticides as honeybees (Goulson *et al.*, 2008), also are parasitized by *N. ceranae* as well as *N. bombi* (Plischuk *et al.*, 2009), and are also declining (Goulson *et al.*, 2008). With the increase in agricultural dependency on pollinators (Aizen *et al.*, 2008) and the pollinator declines looming worldwide, now, more than ever, studies are needed that reveal the interplay between our efforts at insect control, like the use of insecticides, and the pathogens that naturally infect the insect pollinators on which we depend for our survival.

## Experimental procedure

Experiments were performed at the Institut National de la Recherche Agronomique of Avignon (France) with bees that were a mixture of *Apis mellifera ligustica* and *Apis mellifera mellifera* typically used for beekeeping in south-east France. *Nosema* infection and exposure to imidacloprid were performed on 1-day-old bees held in cages (10.5 × 7.5 × 11.5 cm) and in the dark at 28°C and 70% relative humidity. They were fed *ad libitum* with candy (30% honey, 70% powdered sugar) and water. To simulate as much as possible colony rearing conditions, caged bees were also supplied with pollen to provide proteins required for their normal development and exposed to a Beeboost® (Pherotech, Delta, BC, Canada) releasing one queen-equivalent of queen mandibular pheromone per day.

In order to test the interactions between *Nosema* and imidacloprid on mortality and immunity, four experimental groups were created: control group, groups infected with *Nosema*, groups chronically exposed to imidacloprid and groups both infected with *Nosema* and chronically exposed to imidacloprid.

The chronic treatments were performed over 10 days. Indeed, mortality due to artificial rearing might be observed in longer periods. Three cages of 30 bees and two cages of 120 bees per experimental group and colony were, respectively, used for the mortality and immune assays. The experiments were repeated using bees from three colonies. Both mortality and immune assays were performed at the same time to avoid any bias due to the weather or season on bee physiology.

### *Nosema* infection

Spores were isolated from colonies, according to the protocol developed by Higes and colleagues (2007). The spore concentration of the suspension was determined using a haemocytometer, and the solution was used for honey bee infection. To ensure that each bee of *Nosema*-infected groups was infected with the same dose of *Nosema* when starting the experiments, they were fed individually as in Malone and Gatehouse (1998) with 2 µl of a freshly prepared 50% sucrose solution containing 200 000 spores of *Nosema*. Similar spore numbers are known to cause an infection in worker bees (Malone and Gatehouse, 1998; Higes *et al.*, 2007). Control and imidacloprid-treated bees were fed with a sucrose solution.

At days 5 and 10, bees from each cage were collected to determine the level of *Nosema* infection using a haemocytometer. The species identification revealed that our bees were infected with both species of *Nosema*, *N. apis* and *N. ceranae* as it is the case in other regions (Paxton *et al.*, 2007) (see Supporting information for the procedure).

### Imidacloprid treatment

The neonicotinoid imidacloprid [1-(6-chloro-3-pyridylmethyl)-N-nitro-imidazolidin-2-ylidene amine] was present in concentration reaching 5 µg kg^−1^ in honey and pollen in various studies (Bogdanov, 2006), which represents a concentration of around 7 µg kg^−1^ of sugar syrup. Accordingly, low, average and high concentrations corresponding to 0.7, 7 and 70 µg kg^−1^ of imidacloprid were used for the mortality assay. Preliminary results obtained on young bees showed that an imidacloprid concentration of 7 µg kg^−1^ corresponds to a sub-lethal dose in an acute intoxication assay (data not shown).

A stock solution of imidacloprid (Cluzeau, France) was diluted to the required concentration with dimethyl sulfoxide (DMSO), water and finally sucrose feeding to obtain final concentrations of 50% (w/v) sucrose, 0,1% DMSO and imidacloprid at the appropriate concentration (0.7, 7 and 70 µg kg^−1^). The imidacloprid solutions were freshly prepared each day. Solutions containing sucrose and DMSO were used as controls. Bees were chronically exposed to imidacloprid by ingesting imidacloprid-containing sugar syrup (50% sucrose solution, w/v) 10 h per day. This method allowed chronic treatments with minimal disturbance. The feeders were replaced each day at the same time of the day and to estimate the energetic demands the daily sucrose consumption was measured for each cage. The amount of sucrose consumed was expressed per day (10 h period) and per bee, by dividing the amount consumed in a cage by the number of remaining bees in this cage. The rest of the time, they were fed with candy and water *ad libitum*.

### Immune parameters

Immune parameters were measured in 5- and 10-day-old bees. To determine the THC, haemolymph was extracted with micro capillaries (10 µl) from the second abdominal tergite and diluted 2:10 in ice cold ringer saline. Total haemocyte count per microlitre of haemolymph was performed using a phase contrast microscope (×200) with haemocytometer. Phenoloxidase activity was measured on abdomen devoid of its digestive tract instead of haemolymph. The specific PO activity was lower in the abdomen compared with haemolymph but the variability in the activity was also lower in the abdomen (Fig. S1), probably due to a high variance in the volume of haemolymph between individuals. Glucose oxidase is synthesized in the HPGs (Ohashi *et al.*, 1999). As the size of the HPGs reaches a maximum in *c*. 10-day-old bees (Crailsheim *et al.*, 1992), GOX activity was measured at day 10 on whole heads. For each enzyme, the activity was normalized to the protein concentration of each sample. In order to correlate the GOX activity with the size of the HPG, we also dissected HPG from workers of each experimental group and their size was classified into five defined stages of development (stage 1: totally undeveloped, stage 5: fully developed).

### Statistical analysis

In the mortality assay, daily counts of the number of dead bees of corresponding colony replicates were added together. Then, the daily cumulative numbers of dead bees were log-transformed. Analysis of mortality rates was performed using a generalized linear model function. The effects of treatments on *Nosema* infection, THC, protein concentration, enzymatic activity, HPG development and feeding behavior was determined using analysis of the variance (two- and three-way anova and repeated measures two-way anova for the last measurement). Bonferroni post-hoc unpaired *t*-tests were performed for pairwise comparisons between the different treatments. Statistical analyses were performed using Sigmastat 3.10 and Statistica 8.0.

## Supporting information

**Supplementary text 1.***Nosema* species identification.

**Supplementary text 2.** Enzymatic activity measurements.

**Fig. S1.** Specific PO activities in different body parts of honeybees. Phenoloxidase activity was measured in haemolymph, thorax, abdomen and abdomen devoid of the digestive tract. Means ± SE are shown.
