# Supplementary material for: Interactions between Nosema microspores and a neonicotinoid weaken honeybees (Apis mellifera)
Source: Environ Microbiol. 2010 Mar;12(3):774–82. doi: 10.1111/j.1462-2920.2009.02123.x (PMC2847190; doi:10.1111/j.1462-2920.2009.02123.x)
Supplement: Supplementary file 1 [file emi0012-0774-SD1.doc]

**Supporting information**

**Nosema species identification**

To proceed with the species identification, germination of spores was induced for 15 min with 0.3% hydrogen peroxide to get an easier access to the genomic DNA (Higes et al., 2006). Then, in order to release DNA from the cells, 0.3 g of 0.1 mm silica beads (VWR International, Fontenay-sous-Bois, France) were added to germinated spores and the samples were shaken in a cell disruptor (FP120 FastPrep Instrument, MP Biomedical, Illkirch, France) at maximum speed during 45 sec. This step was repeated three times with incubation on ice for 1 min between each run. DNA was directly amplified by PCR using specific primers of the two species *Nosema apis* (For: 5’-ccattgccggataagagagt-3’/ Rev: 5’-cacgcattgctgcatcattgac-3’) and *N. ceranae* (For: 5’-cggataaaagagtccgttacc-3’)/ Rev: 5’-tgagcagggttctagggat-3’) (Chen et al., 2008). Amplification was performed using a GeneAmp PCR system 2400 thermal cycler (Perkin-Elmer, Courtaboeuf, France) and the Expand high fidelity DNA polymerase (Roche Diagnostics, Meylan, France). Negative controls (water instead of germinated spores) were also analyzed. PCR products (about 400 bp) were visualized on 2 % agarose gels and sequenced for further confirmation (GenomeExpress, France). Sequence results were analyzed using the BLAST server in the GenBank database (NCBI, NIH).

**Enzymatic activity measurements**

*Tissues homogenization*

Abdomens and heads were dissected and immediately placed in ice cooled tubes and frozen at -20°C until homogenization. The different biological compartments were homogenized at 4°C, using a TissueLyser (Qiagen, Courtaboeuf, France), in phosphate buffer (80 mM NaH_2_PO_4_/Na_2_HPO_4_, 20 mM NaCl, 1% (w/v) Triton X-100, pH 7.4) containing a mixture of 2 mg/ml of antipain, leupeptin and pepstatin A, 25 units/ml of aprotinin, 0.1 mg/ml of trypsin inhibitor as antiproteolytic agents. The homogenates were then centrifuged at 15,000 g for 20 min at 4°C. The supernatant was used for analysis of enzymatic activities and protein contents.

*PO activity*

PO activity assays are based on the conversion of L-Dopa (3,4-Dihydroxy-L-phenylalanine) to melanin. Freshly extracted samples were combined with extraction buffer (80 mM NaH_2_PO_4_/Na_2_HPO_4_, 20 mM NaCl, 1% (w/v) Triton X-100) prior to enzymatic measurement. Tissue homogenates were diluted using a 2:3 extract ratio. The reaction mixture consisted of 90 μL of distilled water and 20 μL of sodium phosphate buffer (100 mM NaH_2_PO_4_, 200 mM NaCl, pH 7.2), to which 50 μL of freshly extracted sample was added. The microplate was incubated 5 min at 37°C before adding 40 μL of the enzymatic substrate L-Dopa (2 mg/mL) to each well. PO activity was quantified by recording the change in sample absorbance at 490 nm for 10 min. Analysis was repeated three times for each sample. Absorbance data were obtained using a BioTek Synergy HT100 plate reader (BioTek Instruments, Colmar, France).

*GOX activity*

GOX catalyses the conversion of β-D-glucose and O_2_ into D-gluconolactone and H_2_O_2_ (Bentley, 1963). H_2_O_2_, in the presence of o-dianisidine, can in turn be converted by peroxidase into oxidised o-dianisidine, a spectrophotometrically active compound. GOX activity was assayed after adapting the method of (White et al., 1963) to honey bee tissues. The reaction mixture consisted of 100 μL of distilled water and 50 μL of potassium phosphate buffer (500 mM KH_2_PO_4_/K_2_HPO_4_, pH 7.0), containing 20 μL of glucose (100 mM) and 10 μL of horseradish peroxidase (2.5 U). Glucose solution was prepared 1 h in advance to allow β-mutarotation of the glucose and the reaction mixture was incubated for 10 min at 37°C prior to absorbance measurement. 10 μL of freshly homogenized sample was incubated for 10 min at 37°C in the microplate. Then, 20 μL of o-dianisidine (3 mM) was extemporaneously added to the solution. GOX activity was quantified by recording the change in sample absorbance at 430 nm for 1.5 h. Analysis was repeated three times for each sample.

*Protein determination*

The enzymatic activity was normalized to the protein concentration of each sample determined by a standard Bradford protein quantification assay (Bradford, 1976) adapted to microplate. Bovine serum albumine (BSA) was used as standards. Samples were diluted in distilled water and 10 μL of this solution was mixed with 150 μL of distilled water and 40 μL of protein assay dye reagent concentrate. After thorough homogenization, the microplate was incubated for 30 min in obscurity and absorbance was measured at 595 nm. Protein concentrations were determined by using the equation of the linear regression obtained from the standard curve.

*Modeling of PO and GOX kinetic curves*

Enzymatic activities were calculated by modeling the kinetic curves obtained from the measures of absorbance and determining the slope of the steady-rate phase in the kinetics. This data was then normalized to the protein concentration of the sample to estimate specific activities for each enzyme. The specific activities were expressed as variations of milli-unit of absorbance per mg of protein (muA.min^-1^.mg^-1^ of proteins). For PO, a classical enzymatic model adapted to first order reactions was used (Cornish-Bowden, 1995). Kinetic absorbance curves were adjusted according to a time function as follows:


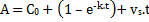


where C_0_ (absorbance at t=0), k (the time constant of the activation process) and v_s_ (the steady-state rate of the activation process) are the constants to estimate. For GOX, the model was adapted from logistic equations (Murray, 2008). This logistic function is suitable to fit the coupled reaction, as it accounts for the delay related to the first reaction step. Kinetic absorbance curves were adjusted according to a time function as follows:


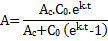


where C_0_ (absorbance at t=0), Ac (maximum absorbance) and k (the time constant of the activation process) are the constants to estimate.

**References**

Bentley, R. (1963) Glucose oxidase. In *The Enzymes*. Boyer, P.D., Lardy, H., and Myrbak, K. (eds). New York: Academic Press, pp. 567-584.

Bradford, M.M. (1976) A rapid and sensitive method for the quantification of microgram quantities of protein utilizing the principle of protein-dye binding. *Anal Biochem* **72**: 248-254.

Chen, Y., Evans, J.D., Smith, I.B., and Pettis, J.S. (2008) *Nosema ceranae* is a long-present and wide-spread microsporidian infection of the European honey bee (*Apis mellifera*) in the United States. *J Invertebr Pathol* **97**: 186-188.

Cornish-Bowden, A. (1995) *Fundamentals of enzyme kinetics*. London: Portland Press.

Higes, M., Martin, R., and Meana, A. (2006) *Nosema ceranae*, a new microsporidian parasite in honeybees in Europe. *J Invertebr Pathol* **92**: 93-95.

Murray, J.D. (2008) *Mathematical biology I. An Introduction*. New York: Springer.

White, J.W.J., Subers, M.H., and Schepartz, A.I. (1963) The identification of inhibine, antibacterial factor in honey, as hydrogen peroxide, and its origin in a honey glucose oxidase system. *Biochem Biophys Acta* **73**: 57-70.

**Figure S1: Specific PO activities in different body parts of honeybees.**

PO activity was measured in hemolymph, thorax, abdomen and abdomen devoid of the digestive tract. Means ± SE are shown.
